# Risk factors for diphtheria outbreak in children aged 1-10 years in East Kalimantan Province, Indonesia

**DOI:** 10.12688/f1000research.16433.1

**Published:** 2018-10-10

**Authors:** Iwan Muhamad Ramdan, Rahmi Susanti, Riza Hayati Ifroh, Reny Noviasty

**Affiliations:** 1 Public Health Faculty, Mulawarman University, Samarinda, East Kalimantan, 75123, Indonesia

**Keywords:** Pediatrics Diphtheria, immunization status, nutrition status, mobility, source of transmission, knowledge and attitude, physical home environment.

## Abstract

**Background**: Diphtheria remains a health problem, especially in developing countries. In November 2017, the Indonesian Ministry of Health stated that there was a diphtheria outbreak in Indonesia. East Kalimantan is one of the provinces that experienced this disease outbreak. This study analyzes the risk factors for diphtheria outbreak in children aged 1-10 years.

**Methods:** A case-control study was conducted on 37 respondents. Research variables consist of immunization status against diphtheria, pertussis and tetanus (DPT), nutritional status, children mobility, source of transmission, physical home environment (natural lighting, ventilation area, occupancy density, wall and floor type), knowledge of diphtheria and attitudes towards the diphtheria prevention program.

**Results:** We found that the most of the children who had diphtheria had been immunized against DPT. Additionally the nutritional status of children (p=0.049), mobility (p=0.000) and the source of transmission (p=0.020) were significantly associated with diphtheria.

**Conclusions:** Child/parent mobility (OR=8.456) is the main risk factor for diphtheria outbreak. It is recommended to limit the mobility of children to travel to areas that are experiencing increased cases of diphtheria, improve the nutritional status, and further research on the effectiveness of diphtheria vaccine.

## Introduction

Although vaccination programs have succeeded in reducing the incidence of diphtheria in the world, diphtheria remains a health problem, especially in the Asian region. The World Health Organization reports that the number of diphtheria in 2013 was 4,680 cases which were widespread and mostly concentrated in the Asian continent, including India (3,313 cases), Indonesia (775 cases), Iran (190 cases), Pakistan (183 cases), and Nepal (103 cases). Indonesia has the second highest number of diphtheria cases, with 775 cases ^1, 2^.

In November 2017, the Indonesian Ministry of Health stated that there was a diphtheria outbreak in Indonesia. This is based on reports from various provincial health offices, with 593 cases documented between 1 January and 1 November 2017. There was a surge in the number of cases. Previously, there were 415 cases in 2016, 502 cases in 2015 and 502 cases in 2014. East Kalimantan is one of the provinces that experienced a diphtheria outbreak, with all cases occurring in children aged 1–10 years ^3^.

Diphtheria, taken from Greek "Diphtera", which means leather hide, was first identified by Hippocrates in the 5 ^th^ century BC ^4^. This disease mostly occurs in children under 5 years of age, but currently occurs in children over 5 years (5–19 years) and in adults ^5^. Several studies have shown that low vaccination coverage, crowding and migration, or a combination of host, agent, and environmental factors, can influence the incidence of diphtheria ^6, 7^. Other factors include nutritional status and parental behavior, personal hygiene of children ^8^, density of house occupancy, humidity in the house, type of floor of the house and the source of transmission (contracting from other people), parents knowledge about diphtheria ^9^, parent education level ^10, 11^, child age, home lighting, and house ventilation ^12^.

This study aims to determine the risk factors for diphtheria outbreaks in children aged 1–10 years in the East Kalimantan province of Indonesia, by involving immunization factors, children's factors, home environmental factors and parents' knowledge and attitude factors.

## Methods

### Study design and settings

A case control study was conducted on 37 respondents (18 cases, children with diphtheria and 19 controls, healthy children), between April to August 2018, located in six districts in the province of East Kalimantan (City of Samarinda, Bontang, Balikpapan and Districts of Kutai Kartanegara, Kutai Timur and Berau). The population approached for recruitment was all children aged 1–10 years with diphtheria recorded in the East Kalimantan provincial health office from January 1, 2017 to March 1, 2018. The study began after the researcher obtained the permission and address of the child suffering from diphtheria from the relevant authorities. Data collection was conducted through visiting the home of each child suffering from diphtheria (case) and neighbors or live close to a case group, and obtaining written informed consent from a parent/guardian.

The case group was formed of children suffering from diphtheria, with inclusion criteria: age 1–10 years, recorded in the East Kalimantan Provincial Health Office register from January 2017–February 2018, residing in the city of Balikpapan, City of Samarinda, City of Bontang, District of Kutai Timur, District of Kutai Kartanegara, and District of Berau, did not move to another area, the house that occupied had not been renovated from 1 week before the child suffering from diphtheria until the data collection, the families of the patients were willing to become respondents and were willing to be interviewed.

The control group was formed of children who did not have diphtheria, with the following inclusion criteria: aged 1–10 years, residing in the City of Balikpapan, City of Samarinda, City of Bontang, District of Kutai Timur, District of Kutai Kartanegara, and District of Berau, being a neighbor of the child with diphtheria/living in one area with a case group, not to move to another area, the house that occupied was not renovated from one week before the neighboring child was suffering from diphtheria until the time of data collection, the children’s family willing to become a respondent and willing to be interviewed.

All children with diphtheria were used as respondents (total sampling), while the control group was obtained using non-random sampling techniques. The control group was recruited by identifying children who met the inclusion criteria that were friends with those in the case group or lived nearby.

The dependent variable in this study was diphtheria, while the independent variables consisted of age, gender, DPT immunization status, nutritional status, childhood mobility (a travel history to an area that is experiencing in cases of diphtheria), source of transmission (friends at school or neighbors who are experiencing of diphtheria), the house’s physical environment (natural lighting, house ventilation, occupancy density, type of wall and floor), knowledge of diphtheria and attitude towards the diphtheria prevention program.

### Data collection and measurement

Administered structured questionnaire and an observation checklist were used to collect data. The questionnaire and observation checklist used in this study consists of eight sections. Section A: Socio demographic information (initial name, place and date of birthaddress); Section B: Immunization status (data obtained by interview and confirmed by the immunization card for each child); Section C: Nutritional status (height and weight of the children, then calculation of body mass index); Section D: physical home environment (natural lighting in the house and bedroom, the width of the house ventilation, the floor area of the house, the number of people sleeping in a room with children suffering from diphtheria, the type of house wall, the type of house floor); Section E: Source of transmission (history of direct contact with a friend suffering from diphtheria in a home environment or at school); Section F: Mobility (history of child traveling/staying outside the city of domicile, one week before illness); Section G: Knowledge of diphtheria (causes, signs and symptoms, modes of transmission, benefits of DPT immunization, other prevention methods); Section H: attitude against diphtheria prevention program (favorable or unfavorable). Dataset 1 contains all de-identified responses to the questionnaire ^13^.

To reduce interview bias, researchers provide adequate explanations before the interview begins, motivated respondents to give honest answers, questionnaires are arranged in simple language and easily understood and provides sufficient time for interviews. The determination of DPT immunization status, nutritional status and healthy housing standards are in line with those described by the Indonesian Health Ministry regulations ^14– 16^.

### Statistical analysis

Data were analyzed using chi square and multiple logistic regression. To see the risk factors related to Diphtheria, an odds ratio (OR) with a 95% confidence interval was calculated. Data analysis using the Statistical Package for the Social Sciences (SPSS ver. 21, Chicago, IL, USA).

### Ethical approval

The study was reviewed and approved by the Ethical Commission of Health and Medical Research, Faculty of Medicine, Mulawarman University Indonesia, (approval number: 42/KEPK-FK/V/2018), which refers to The International Ethical Guidelines for Biomedical Research Involving Human Subjects and The international ethical guidelines for epidemiological studies, from Council for International Organizational Organizations of Medical Sciences (CIOMS 2016). Informed written consent was obtained from a parent or guardian of the participants prior to their participation. The informed consent stated the purpose of the study, data confidentiality, and the voluntary right of participation in the study, as well as provided the guarantee that no participant suffered any harm as a result of his/her participation in the study.

## Results

### Variables

The sex of the case group was mostly male (66.6%), age was mostly > 5–10 years (66.6%), DPT immunization status was mostly complete (83.3%), nutritional status was mostly bad (72.2%), mobility of the children was mostly “yes” (61.15%), source of contamination was mostly “no” (77.7%), knowledge of diphtheria was balanced between good and bad (50%), attitude towards the diphtheria prevention program was mostly favorable (55.5%), wide of home ventilation was mostly bad (77.7%), home density of occupancy was mostly good (72.2%), home wall type was mostly made from concrete brick without plastering (61.1%) and home floor type was mostly ceramics (66.6%).

The sex of the control group were mostly male (52.6%), the age was mostly 1–5 years (52.6%), DPT immunization status was mostly complete (63.1%), nutritional status was mostly good (63.1%), mobility of the children was mostly “yes” (84.2%), source of contamination was mostly “yes” (63.1%), knowledge of diphtheria was mostly good (52.6%), attitude towards the diphtheria prevention program was mostly favorable (52.6%), wide of home ventilation was mostly bad (68.4%), home density of occupancy was mostly good (63.1%), home wall type was mostly made from concrete brick without plastering (57.8%) and home floor type was mostly ceramics (63.1%) ( Table 1 and Table 2).

**Table 1.** Characteristics of respondents (n=37).

Characteristics	Cases		Control		Total	
	n	%	n	%	n	%
Gender						
Male	12	66.6	10	52.6	22	59.4
Female	6	33.3	9	47.3	15	40.5
Age, years						
1–5	6	33.3	10	52.6	16	43.2
>5–10	12	66.6	9	47.3	21	56.7
DPT immunization status						
Complete	15	83.3	12	63.1	27	72.9
Incomplete	3	16.6	7	36.8	10	27.0
Nutritional status						
Good	5	27.7	12	63.1	21	56.7
Bad	13	72.2	7	36.8	16	43.2

**Table 2.** Results of bivariate analysis.

Risk factor	Cases		Control		P-value	OR (95% CI)
	N	%	N	%		
Age, years					0.325	0.450 (0.119–1.703)
1–5	6	33.3	10	52.6		
>5–10	12	66.6	9	47.3		
Gender					0.508	1.800 (0.476–6.812)
Male	12	66.6	10	52.6		
Female	6	33.3	9	47.3		
DPT immunization status					0.269	0.343 (0.073–1.617)
Complete	15	83.3	12	63.1		
In complete	3	16.6	7	36.8		
Nutritional status					0.049	4.457 (1.11–17.89)
Good	9	50	12	63.1		
Bad	9	50	7	36.8		
Mobility					0.000	6.800 (2.253–31.645)
Yes	11	61.1	16	84.2		
No	7	38.8	2	10.5		
Source of contamination					0.020	0.167 (0.039–0.711)
Yes	4	22.2	12	63.1		
No	14	77.7	7	36.8		
Knowledge parent					1.000	1.111 (0.306–4.037)
Good	9	50	10	52.6		
Bad	9	50	9	47.3		
Attitude towards immunization program					1.000	0.889 (0.244–3.243)
Favorable	10	55.5	10	52.6		
Unfavorable	8	44.4	9	47.3		
Wide of home ventilation					0.714	1.615 (0.370–7.049)
Good (>10%)	4	22.2	6	31.5		
Bad (<10%)	14	77.7	13	68.4		
Home density of occupancy					1.000	0.833 (0.203–3.427)
Good (>8 m ^2^/person)	13	72.2	12	63.1		
Bad (<8 m ^2^/person)	5	27.7	7	36.8		
Home wall type					1.000	1.143 (0.307–4.254)
Plastering concrete brick	7	38.8	8	42.1		
Concrete brick without plastering	11	61.1	11	57.8		
Home floor type					1.000	1.167 (0.302–4.512)
Concrete plastering	6	33.3	7	36.8		
Ceramics	12	66.6	12	63.1		

### Analysis of the variables

The results of the bivariate test showed that nutritional status (p=0.049) (OR=4.457), mobility (p<0.001) (OR=6.812) and source of transmission (p=0.020) (OR=0.16) were significantly associated with the incidence of diphtheria in East Kalimantan Province, Indonesia ( Table 2).

Multivariate analysis performed on the variables which proved to be significantly associated with the incidence of diphtheria, i.e. nutritional status, mobility and source of transmission. The results show that mobility variables (OR=8.456) is the main risk factor for diphtheria in East Kalimantan Province. ( Table 3).

**Table 3.** Results of logistic regression for risk factors of diphtheria.

Risk factors	Adjusted OR	95% CI	P-value
Nutritional status	0.810	0.065–10.073	0.870
Source of contamination	0.134	0.012–1.519	0.105
Mobility	8.456	5.643–12.672	0.001

OR, odds ratio; CI, confidence interval.

All raw data and demographic information obtained from subjects during the present study Click here for additional data file. Copyright: © 2018 Muhamad Ramdan I et al. 2018 Data associated with the article are available under the terms of the Creative Commons Zero "No rights reserved" data waiver (CC0 1.0 Public domain dedication).

## Discussion

The results of univariate analysis demonstrated that most patients with diptheria had received complete DPT immunization. The result of bivariate analysis revealed no correlation between DPT immunization status and diphtheria infection. This result is notable, and indicates that further investigation is required on the effectiveness and potential of vaccines. A further example documented by Ningtyas *et al*. ^17^, concerning cases of measles in children in Indonesia, also concluded that the incidence of measles in children remained high in areas with high measles immunization coverage; however, this was related to the effectiveness of vaccine quality due to health worker skill factors in providing vaccines and availability of vaccine facilities. Other studies have documented the variable thermolability of vaccines, caused by breaks in the cold chain, can lead to loss of vaccine potency ^18^. The results of this study complement the findings of Dhinata *et al*. ^19^, which found no correlation between patient immunization status and severity, or fatality of diphtheria in the Sampang District of Indonesia.

Complete immunization status does not guarantee the child is free from the risk of diphtheria. Sadoh and Sadoh ^20^ concluded that two out of three children with diphtheria in Nigeria had been completely immunized against DPT, and suggested the use of DT boosters in developing countries. Previously, Gowin *et al*. ^21^ proved that even though tetanus and diphtheria antibody concentrations are quite high in children that have been immunized, the percentage of children protected against diphtheria is smaller than those against tetanus. Likewise, the results of research by Phadke *et al*. ^22^, revealed that several pertussis outbreaks in United States also occurred in highly vaccinated populations, and indicating waning immunity.

We found the nutritional status of children was significantly associated with the incidence of diphtheria. The results of this study are consistent with other studies that concluded nutritional status associated with increased risk and/or severity of infections disease ^23^; Children's nutritional status is significantly associated with diphtheria in Situbondo Indonesia ^24^, Children's nutritional status and immune deficiencies reduce the body's response to vaccines ^25, 26^. The implications of this finding are, to reduce the risk of the occurrence of diphtheria in children, the improvement of nutrition is absolutely necessary.

The results prove that the mobility of respondents (travel history to an area that is experiencing a surge in cases of diphtheria) is significantly related to the incidence of diphtheria, this result is consistent with other studies by Patil *et al*. ^27^ which concludes the mobility creates a vulnerability of pediatrics diphtheria outbreak in district of central India. Population migration increases the risk of transmission of infectious diseases ^28^, transmission of measles, rubella, diphtheria, tetanus, polio and *Haemophilus influenzae* is strongly influenced by population mobility ^29^. High mobility, poor living conditions, and barriers to accessing healthcare are risk factors to facilitate the spread of infectious diseases such as tuberculosis (active and latent), HIV, hepatitis B, hepatitis C, measles, mumps, rubella, diphtheria, tetanus, pertussis, *H. influenzae* type b, strongyloidiasis and schistosomiasis ^30^. Based on this conclusion, the prohibition or limitation of children/parents visiting areas that are experiencing diphtheria outbreaks should be recommended so that the risk of transmission is reduced

## Conclusion

Nutritional status, child mobility and source of transmission were significantly associated with diphtheria. Most children who had diphtheria (83.3%) had received complete immunization of DPT. Mobility of children is the main risk factor of diphtheria. It is recommended to forbid children/parents to visiting the area where a diphtheria outbreak is occurring, and to improve the condition of the child's nutritional status. Further research is needed on the effectiveness of diphtheria vaccine in East Kalimantan Province, Indonesia.

## Data availability

The data referenced by this article are under copyright with the following copyright statement: Copyright: © 2018 Muhamad Ramdan I et al.

Data associated with the article are available under the terms of the Creative Commons Zero "No rights reserved" data waiver (CC0 1.0 Public domain dedication). 

**Dataset 1. All raw data and demographic information obtained from subjects during the present study.** DOI: https://doi.org/10.5256/f1000research.16433.d220825 ^13^.
