# Supplementary material for: Risk factors for diphtheria outbreak in children aged 1-10 years in East Kalimantan Province, Indonesia
Source: F1000Res. 2018 Oct 10;7:1625. [Version 1] doi: 10.12688/f1000research.16433.1 (PMC6844134; doi:10.12688/f1000research.16433.1)
Supplement: Peer review history

# Peer review: Reviewer response for version 1

**DOI:** 10.5256/f1000research.17956.r42801

**Published:** 2019-11-06

**Authors:** Yves Buisson

**Affiliations:** 1 Institut de la Francophonie pour la Médecine Tropicale (IFMT), Vientiane, Lao People's Democratic Republic

This is an interesting, well-structured study analyzing the risk factors for contracting diphtheria in Indonesian children. The article is well written but some improvements are needed before indexing:

1. What were the criteria for defining cases of diphtheria? Only clinically or after bacteriological confirmation?
2. Specify the age group >5-10 years: is it 5-10, or >10, or both (>5)?
3. In "Methods - statistical analysis", it must be stipulated that only the parameters giving a p <0.05 are entered in the logistic regression.
4. The results obtained (cases better vaccinated than the controls and better nutritional status in the controls than in the cases) should lead to discuss more deeply the possibility of a weaker immune response among the cases and suggest a complementary study with dosage of post- vaccine antibodies.
5. A source of contamination was found among the controls, not among the cases; it is in contradiction with the fact that among diphtheria cases, mobility in a region experiencing a recrudescence of diphtheria proves to be the main risk factor. Such discrepancy should be analysed in the discussion.

I confirm that I have read this submission and believe that I have an appropriate level of expertise to confirm that it is of an acceptable scientific standard.

# Peer review: Reviewer response for version 1

**DOI:** 10.5256/f1000research.17956.r39315

**Published:** 2018-10-19

**Authors:** Soedjajadi Keman

**Affiliations:** 1 Faculty of Public Health, Airlangga University, Surabaya, Indonesia

This research article is very good. However, please complete the conclusion in the Abstract: Child/parent mobility (OR=8.456) is the main risk factor for diphtheria outbreak and the nutritional status of children and the source of transmission as well.

 The keywords, introduction, methods, results (including statistical analysis), discussion, conclusion and references are okay.

 Additional comments:

 1.         The study design is appropriate and the work is technically sound.

 2.         The method is quite detailed and analysis provided is absolutely perfect to allow replication by other researchers.

 3.         The statistical analysis is correct and its interpretation is also appropriate.

 4.         The conclusions drawn should be: the main risk factors for diphtheria outbreak are children mobility, source of transmission, and nutritional status. It is recommended to the parents to limit mobility of their children to areas that are experiencing increased cases of diphtheria and improve their childrens’ nutritional status as well. It is recommended for further study to analyze the effectiveness of diphtheria vaccines since both study and control group have already got diphtheria vaccination.

I confirm that I have read this submission and believe that I have an appropriate level of expertise to confirm that it is of an acceptable scientific standard.
